# Unveiling ADAMTS12: A key driver of bladder cancer progression *via* COL3A1-Mediated activation of the FAK/PI3K/AKT signaling pathway

**DOI:** 10.1016/j.jbc.2025.108155

**Published:** 2025-01-04

**Authors:** Jian-hua Xiao, Li-zhe Xu, Jin-zhuo Ning, Fan Cheng

**Affiliations:** 1 Department of Urology, Renmin Hospital of Wuhan University, Wuhan, Hubei Province, P.R. China; 2 Department of Urology, The First College of Clinical Medical Science, China Three Gorges University & Yichang Central People's Hospital, Yichang, Hubei Province, P.R. China

**Keywords:** ADAMTS12, COL3A1, bladder cancer, FAK/PI3K/AKT pathway, growth, apoptosis

## Abstract

Bladder cancer (BCa) is a common and lethal disease characterized by high recurrence rates and limited treatment options. Understanding the molecular pathways of BCa progress is crucial for investigating more effective targeted therapies. While ADAMTS12 is known to contribute to cancer progression and treatment resistance, its prognostic significance and underlying mechanisms in BCa remain poorly understood. To elucidate the molecular pathways and functions of ADAMTS12 in BCa, we employed various experimental approaches, including Transwell invasion assays, flow cytometry analysis, wound-healing assays, CCK-8 assays, and a xenograft tumor model. Our results demonstrated that overexpression of ADAMTS12 significantly enhanced cell growth, migration, and invasion while inhibiting apoptosis through the activation of the FAK/PI3K/AKT signaling pathway. Conversely, the knockdown of ADAMTS12 produced the opposite effects. *In vivo* studies further confirmed that the inhibition of ADAMTS12 effectively suppressed tumor progression. Comprehensive bioinformatics analysis of the TCGA-BLCA dataset and protein-protein interaction networks revealed a strong positive correlation between COL3A1 and ADAMTS12, identifying COL3A1 as a potential downstream target of ADAMTS12. Additionally, we observed a significant increase in the expression levels of ADAMTS12 and COL3A1 in BCa tissues compared to healthy tissues, as confirmed by Western blotting and qRT-PCR analysis. Notably, inhibition of COL3A1 reversed the enhanced cell growth and invasion associated with ADAMTS12 overexpression and suppressed cell apoptosis. Our findings suggest that ADAMTS12 promotes BCa progression through the FAK/PI3K/AKT signaling pathway by regulating COL3A1, highlighting its potential as a valuable marker for diagnosis and prognosis in BCa.

Bladder cancer (BCa) is one of the most prevalent malignant tumors of the urinary system, affecting approximately 613,000 patients and causing about 220,000 deaths annually. It ranks ninth in terms of incidence and 13th in terms of mortality among all malignancies globally (1, 2). In China, the annual incidence of BCa is 80.5 cases per 100,000 individuals, accompanied by a corresponding mortality rate of 32.9 deaths per 100,000 population (3). BCa can be categorized into two subtypes depending on tumor invasion depth, namely, Muscle Invasive BCa (MIBC) and Non-Muscle Invasive BCa (NMIBC). Individuals with NMIBC demonstrate recurrence rates ranging from 31% to 78%, while approximately 10 to 15% of these individuals progress to MIBC (4). Requiring frequent monitoring, transurethral resection, and therapeutic intervention owing to its elevated rate of recurrence and the potential for progression to MIBC, BCa stands out as having the highest lifetime treatment costs among all types of cancer (5). The clinical management of BCa has witnessed limited progress over the past 3 decades, with surgical removal and chemotherapy remaining as the primary treatment options. BCa commonly undergoes a first-line chemotherapy regimen comprising a combination of gemcitabine and cisplatin (6). However, the objective remission rate for patients with advanced BCa who underwent this chemotherapy regimen was only 40 to 60%, and there was a mere 5% improvement in overall survival (7). Consequently, it is imperative to comprehend the molecular pathways underlying BCa progress to investigate targeted therapies with enhanced efficacy.

The ADAMTS (a disintegrin and metalloproteinase with thrombospondin-like motifs) family, consisting of 19 well-known proteins, functions as an extracellular multifunctional enzyme group involved in diverse pathological conditions, including platelet disorders, osteoarthritis, and malignancies (8, 9). As an ADAMTS family member, ADAM metallopeptidase with thrombospondin type 1 motif 12 (ADAMTS12) possesses the potential to modulate tissue remodeling and facilitate cell migration, suggesting its possible involvement in regular inflammatory reactions (10). A prior investigation has shown that the excessive ADAMTS12 expression significantly improves the growth and migration of cells in colorectal malignancy *in vitro*, perhaps *via* strengthening the traditional Wnt signaling pathway (11). Jiang *et al.* (12) reported that ADAMTS12 increases resistance to oxaliplatin treatment in gastric cancer cells by affecting the MAPK/VEGF pathway and is connected with vascular abnormalities, poor survival results, and chemotherapy resistance in gastric cancer patients. Li *et al.* (13) have also observed that ADAMTS12 facilitates cell growth, migration, and invasion in squamous cell carcinoma in the head and neck. Nevertheless, the precise prognostic implications and potential pathways underlying the carcinogenic impacts of ADAMTS12 in BCa are still fully elucidated.

In our recent study, we specifically focused on ADAMTS12 to elucidate its roles in the progression and development of BCa, aiming to ascertain the therapeutic potential of targeting ADAMTS12 for BCa treatment.

## Results

### ADAMTS12 expression is significantly upregulated in BCa

**Figure 1:**
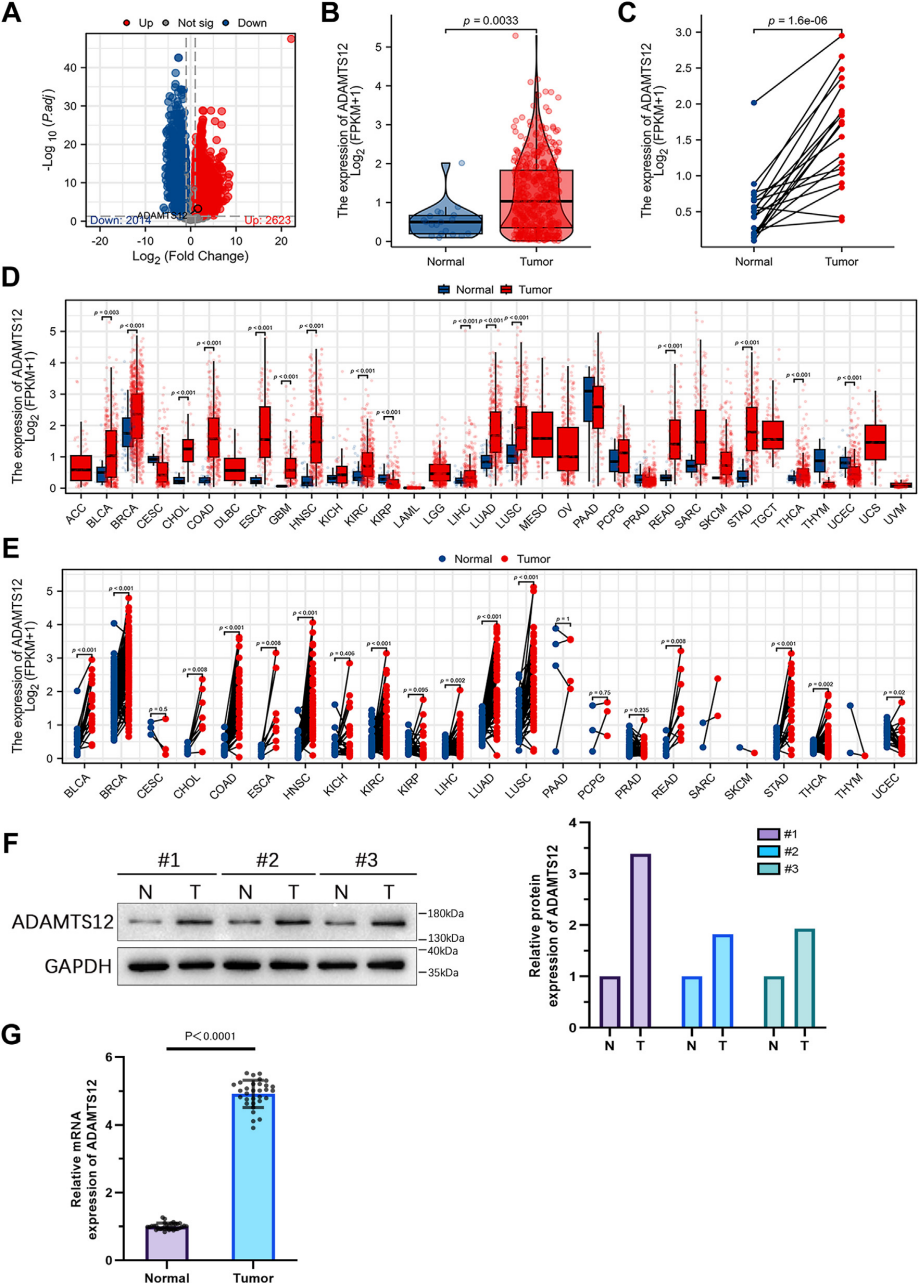
**ADAMTS12 expression is significantly upregulated in BCa.***A*, DEGs in the TCGA-BLCA dataset were determined employing a Volcano plot. *B* and *C*, ADAMTS12 mRNA expression levels in BCa and healthy tissues were examined employing data from TCGA-BLCA. *D* and *E*, ADAMTS12 expression in various malignancy types was examined utilizing data from the TCGA. *F*, the levels of ADAMTS12 in BCa samples and nearby healthy tissues were analyzed employing western blotting (n = 3). *G*, the levels of ADAMTS12 in BCa samples and nearby healthy tissues were analyzed employing qRT-PCR (n = 31). The data are presented as the mean ± SD. Unpaired *t* tests were employed to compare two groups. ∗*p* < 0.05; ∗∗*p* < 0.01; ∗∗∗*p* < 0.001; ns, no statistical difference.

The volcano plot displays the expression distributions of 4637 DEGs identified from a pool of 18,814 genes, using a threshold set at |log_2_FC| > 1. The dataset utilized for this analysis is TCGA-BLCA (Fig. 1*A*). TCGA data evaluation showed a significant increase in ADAMTS12 expression in BCa tissues compared with tissues that were healthy (Fig. 1, *B* and *C*). ADAMTS12 levels were significantly raised in various types of cancer in contrast to the control tissues (Fig. 1, *D* and *E*). As demonstrated by Western blotting and qRT-PCR, ADAMTS12 expression levels were significantly raised in BCa tissues in contrast to tissues that were healthy (Fig. 1, *F* and *G*).

**Figure 2:**
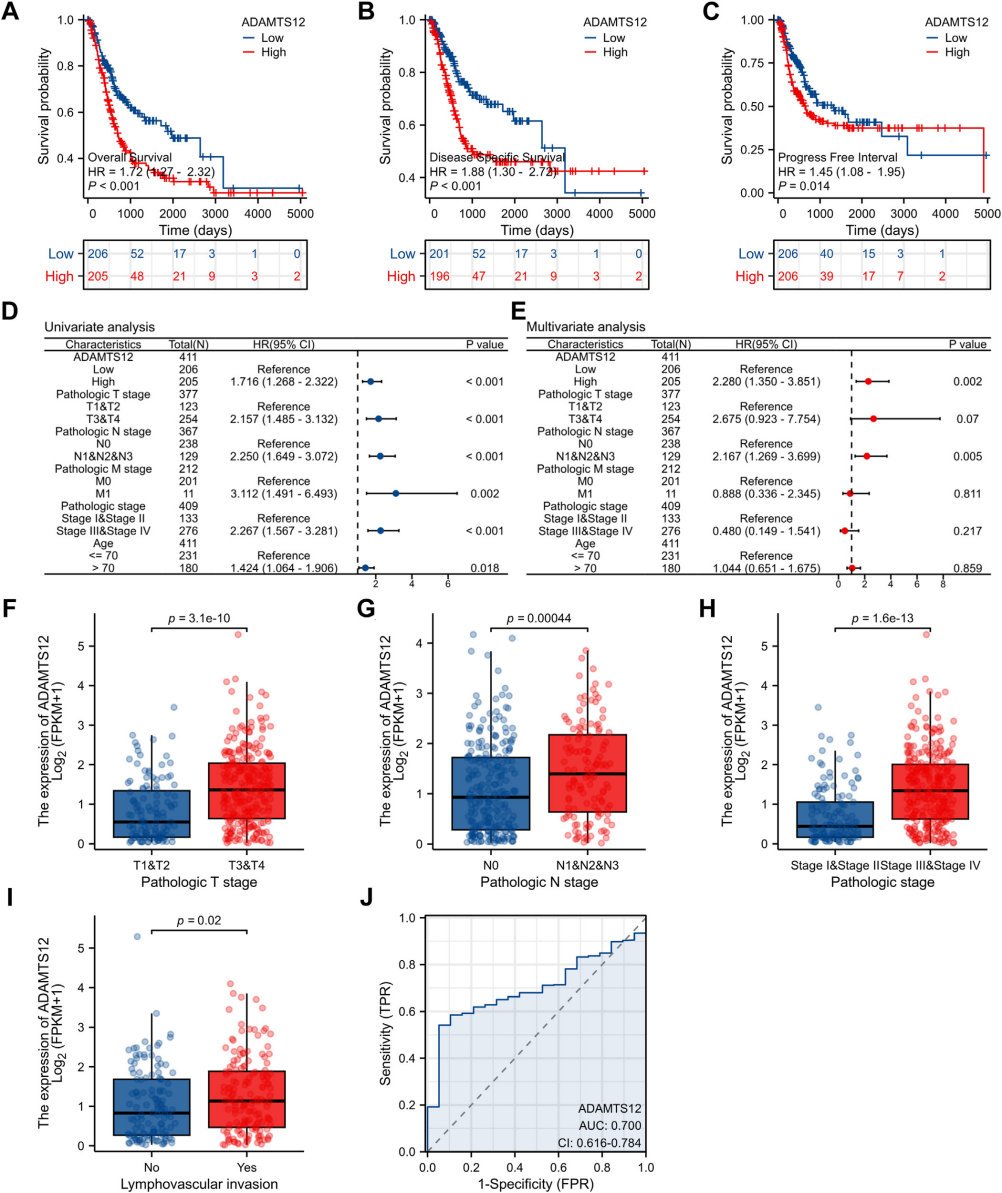
**Comprehensive evaluation of ADAMTS12 in BCa for prognostic assessment, clinicopathological factors analysis, and diagnostic significance determination.***A–C*, the survival curves for OS, DSS, and PFI in patients with BCa were analyzed based on ADAMTS12 expression levels using the TCGA-BLCA dataset. *D* and *E*, the prognostic parameters of OS in the TCGA-BLCA dataset underwent both univariate and multivariate analyses. *F–I*, the relationship between ADAMTS12 expression levels and T stage, N stage, pathological stage, and lymphovascular invasion was investigated in the TCGA-BLCA dataset. *J*, the potential of ADAMTS12 as a diagnostic tool to distinguish between BCa and healthy tissues was investigated utilizing ROC curve analysis. The data are presented as the mean ± SD. Unpaired t-tests were employed to compare two groups. ∗*p* < 0.05; ∗∗∗*p* < 0.001.

The Kaplan–Meier (KM) survival curves revealed a noteworthy correlation between increased levels of ADAMTS12 and reduced disease-specific survival (DSS), progression-free interval (PFI), in addition to overall survival (OS) outcomes among individuals diagnosed with BCa (Fig. 2, *A*–*C*). Additionally, we observed that the expression of ADAMTS12 exhibited significant prognostic value in both univariate and multivariate Cox regression analyses when compared to T, N, and M stages and Age (Fig. 2, *D* and *E*). Additionally, our analysis of the TCGA database revealed significant associations between ADAMTS12 expression and various clinical characteristics, including T, N, and pathological stages and lymphovascular invasion (Fig. 2, *F*–*I*). The predictive performance of ADAMTS12 in the detection of BCa was assessed through ROC analysis, yielding an estimated AUC value of 0.7 (Fig. 2*J*).

### ADAMTS12 facilitates the growth and inhibits the BCa cells' apoptosis

**Figure 3:**
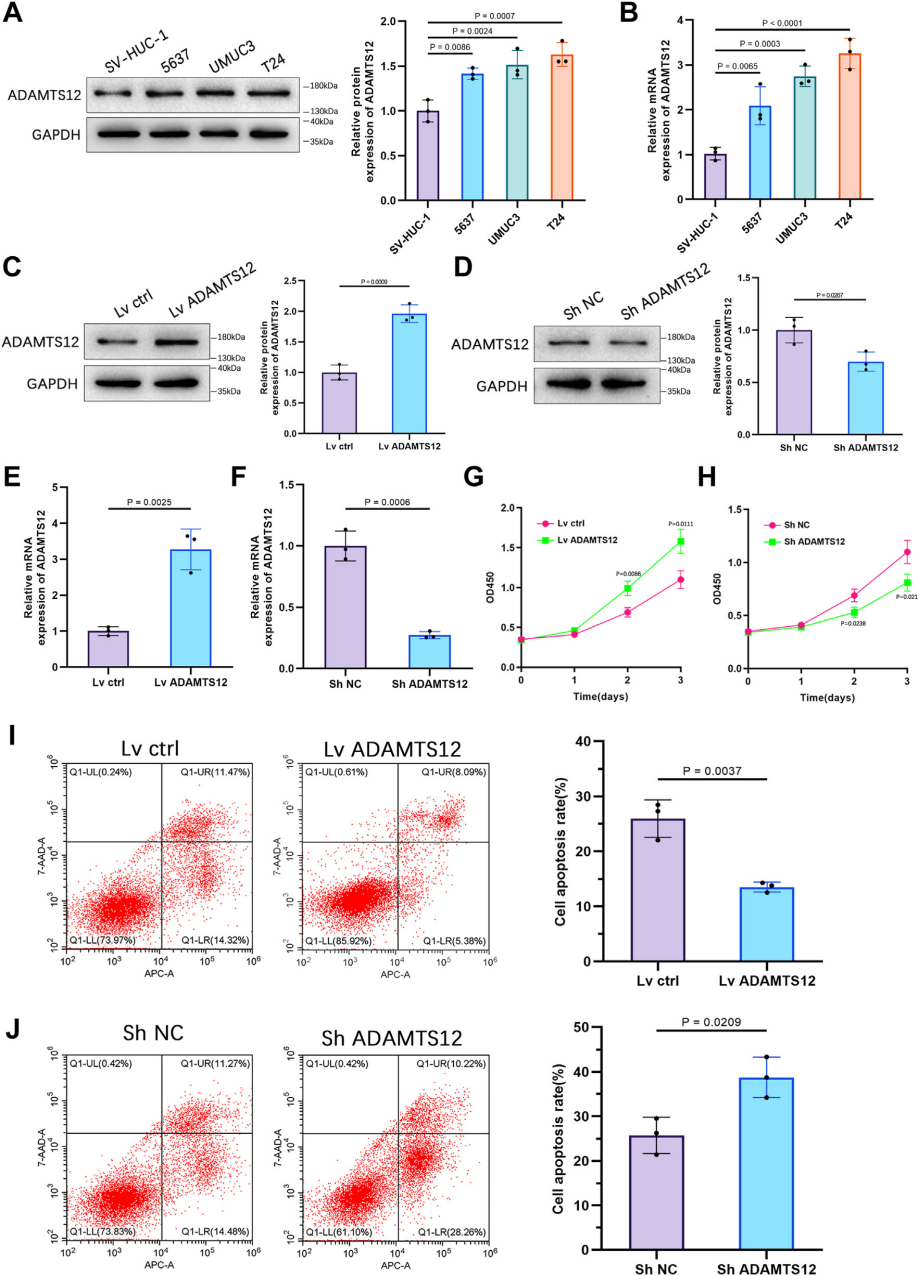
**ADAMTS12 facilitates the growth and suppresses the apoptosis of BCa cells.***A* and *B*, ADAMTS12 levels were assessed in BCa cell lines (T24, UMUC3, 5637) and a non-cancerous cell line (SV-HUC-1) utilizing qRT-PCR and western blotting techniques (n = 3 respectively). *C–F*, the effects of ADAMTS12 overexpression and knockdown in T24 cells were evaluated using Western blotting and qRT-PCR analysis for validation purposes. *G* and *H*, T24 cell viability was examined employing the CCK-8 assay after knockdown and overexpression of ADAMTS12, respectively (n = 3 respectively). *I* and *J*, the apoptotic rate of cells was assessed by flow cytometry following knockdown and overexpression of ADAMTS12, respectively (n = 3 respectively). The data are presented as the mean ± SD. Unpaired t-tests were employed to compare the two groups. One-way ANOVAs with the Tukey test were used for comparisons involving more than two groups. ∗*p* < 0.05; ∗∗*p* < 0.01.

Upregulation of ADAMTS12 was observed in BCa cell lines T24, UMUC3, and 5637 cells in contrast to healthy epithelial cells of the bladder (SV-HUC-1) when assessing the expression of ADAMTS12 using western blotting and qRT-PCR (Fig. 3, *A* and *B*). T24 cell line was detected for consequent experiments owing to its highest expression of ADAMTS12. To explore the effects of ADAMTS12 in BCa cells, we generated stable T24 cell lines with enhanced or reduced levels of ADAMTS12 expression. The effectiveness of both upregulation and downregulation was validated through Western blot analysis and qRT-PCR (Fig. 3, *C*–*F*). CCK-8 assays revealed that the absence of ADAMTS12 significantly impeded, while ADAMTS12 overexpression greatly improved BCa cell proliferation (Figs. 3, *G* and *H* and S1*A*). ADAMTS12's impact on BCa cell apoptosis was evaluated using flow cytometry, revealing that BCa cells overexpressing ADAMTS12 exhibited a significantly suppressed apoptotic rate of cells in contrast to the negative control. In contrast, ADAMTS12-deficient cells demonstrated a substantial increase in apoptotic rate (Figs. 3, *I* and *J* and S1*C*).

### ADAMTS12 promotes migration and invasion in BCa cells

**Figure 4:**
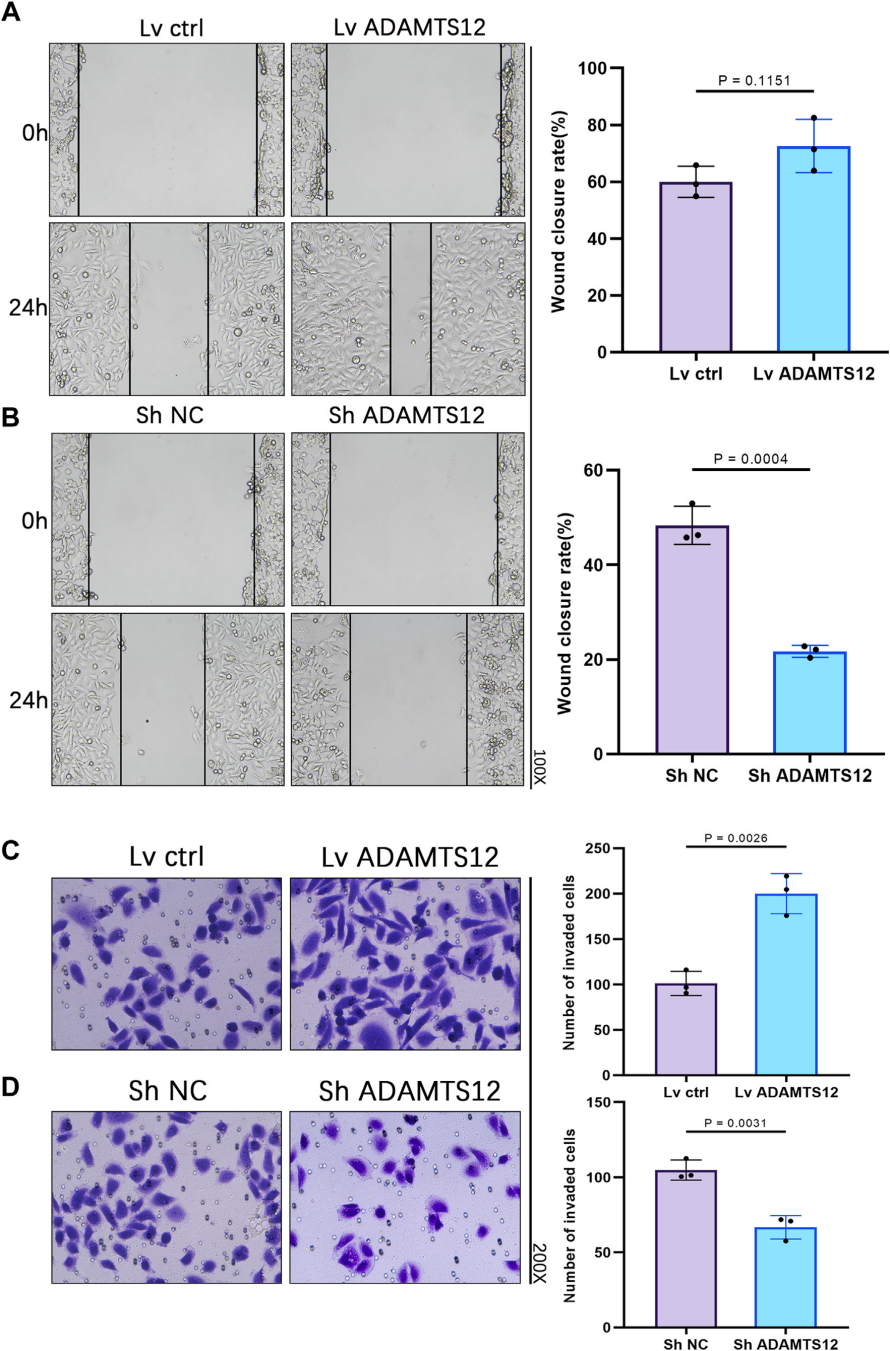
**ADAMTS12 promotes migration and invasion in BCa cells.***A* and *B*, Wound-healing assay demonstrated that the upregulation of ADAMTS12 expression in T24 cells promoted cell migration, whereas the downregulation of ADAMTS12 expression in T24 cells inhibited cell migration (n = 3 respectively). *C* and *D*, transwell assay demonstrated that ADAMTS12 overexpression in T24 cells significantly enhanced invasion, whereas ADAMTS12 expression knockdown in T24 cells effectively suppressed invasion (n = 3 respectively). The data are presented as the mean ± SD. Unpaired *t* tests were employed to compare two groups. ∗*p* < 0.05.

ADAMTS12 overexpression was observed to enhance BCa cell migration in wound healing experiments, whereas a significant ADAMTS12 expression decline attenuated this influence (Figs. 4, *A* and *B* and S1*B*). Transwell assay revealed similar trends, indicating that ADAMTS12 overexpression significantly improved the invasive potential of BCa cells, while ADAMTS12 knockdown suppressed cell invasion (Figs. 4, *C* and *D* and S1*D*).

### TCGA-BLCA dataset and functional enrichment analysis were utilized for the identification of ADAMTS12-associated genes

**Figure 5:**
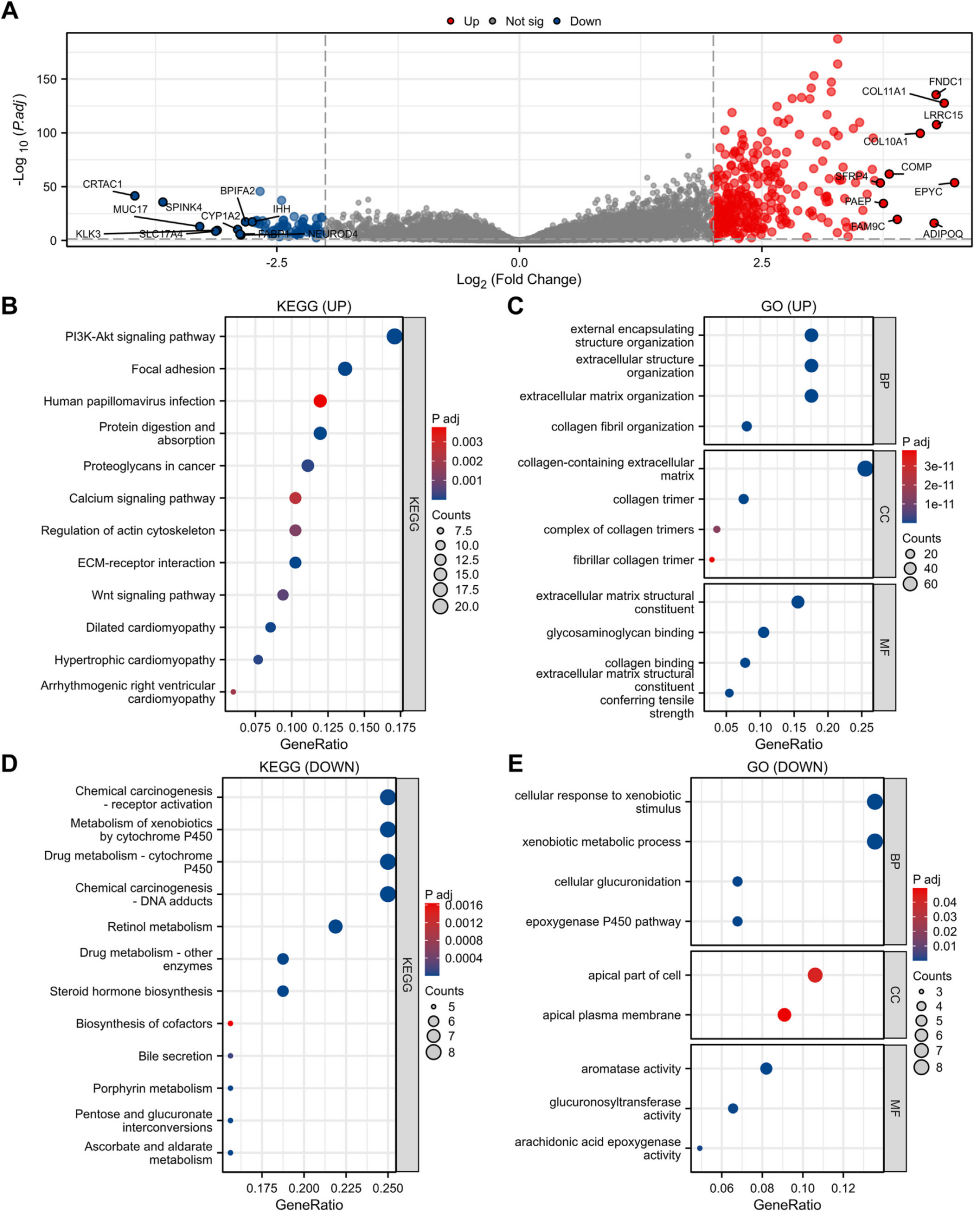
**ADAMTS12-associated genes identification using the TCGA-BLCA dataset and functional enrichment analysis.***A*, the TCGA-BLCA dataset was utilized to identify genes associated with ADAMTS12 using the volcano plot. *B* and *C*, the genes that exhibited a positive correlation with ADAMTS12 in BCa were subjected to comprehensive KEGG and GO analyses. *D* and *E*, the genes that showed a negative correlation with ADAMTS12 in BCa were analyzed using KEGG and GO analyses. GO analysis included biological process (BP), cellular component (CC), and molecular function (MF).

The TCGA-BLCA dataset was utilized to identify genes that exhibited a strong correlation with ADAMTS12 in BCa, specifically focusing on those displaying significant up-regulation or down-regulation with |log_2_FC| > 2 (Fig. 5*A*). The Kyoto Encyclopedia of Genes and Genomes (KEGG) and Gene Ontology (GO) were utilized to thoroughly analyze the genes to conduct a comprehensive investigation. The KEGG analysis suggested a significant enrichment of up-regulated genes primarily connected with the pathway of the PI3K/AKT signaling and focal adhesion (Fig. 5*B*). GO analysis further exhibited that the genes that were upregulated were predominantly enriched in biological mechanisms connected with the extracellular matrix containing collagen (Fig. 5*C*). The KEGG analysis revealed a significant enrichment of down-regulated genes primarily connected with chemical carcinogenesis—receptor activation, while the GO analysis suggested that these downregulated genes were predominantly enriched in processes related to cellular response to xenobiotic stimulus (Fig. 5, *D* and *E*).

### ADAMTS12 modulates the FAK/PI3K/AKT pathway in BCa cells

**Figure 6:**
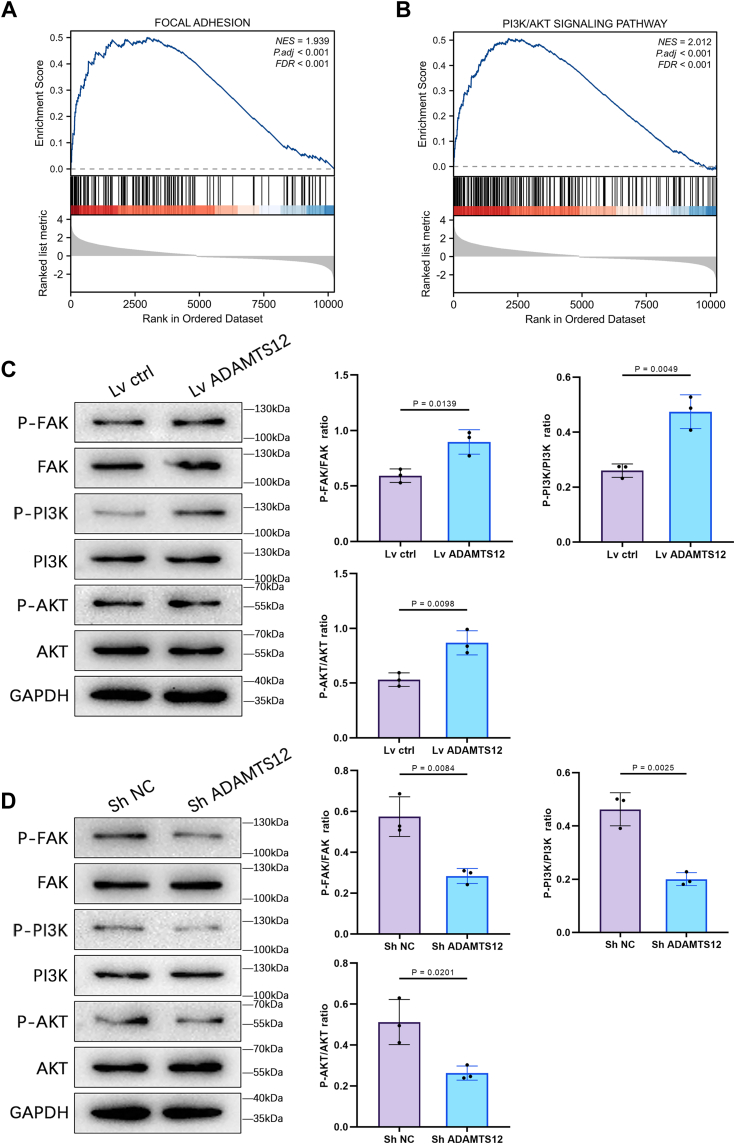
**ADAMTS12 modulates the FAK/PI3K/AKT pathway in BCa cells.***A* and *B*, GSEA analysis demonstrated a significant link between ADAMTS12 expression and focal adhesion, along with the stimulation of the PI3K/AKT signaling pathway. *C* and *D*, -FAK, FAK, p-PI3K, PI3K, p-AKT, and AKT protein levels were assessed in T24 cells following knockdown and overexpression of ADAMTS12 (n = 3 respectively). The data are presented as the mean ± SD. Unpaired *t* tests were employed to compare two groups. ∗*p* < 0.05.

The gene set enrichment analysis (GSEA) exhibited a notable enrichment of Focal adhesion and pathway of PI3K/AKT signaling in the BCa samples with elevated ADAMTS12 expression (Fig. 6, *A* and *B*). The Western blot outcomes exhibited that ADAMTS12 overexpression resulted in p-FAK, p-PI3K, and p-AKT expression upregulation while having no impact on the overall FAK, PI3K, and AKT levels. Additionally, silencing ADAMTS12 caused a decrease in p-FAK, p-PI3K, and p-AKT expression (Fig. 6, *C* and *D*).

### Regulation of COL3A1 expression in BCa cells is mediated by ADAMTS12

**Figure 7:**
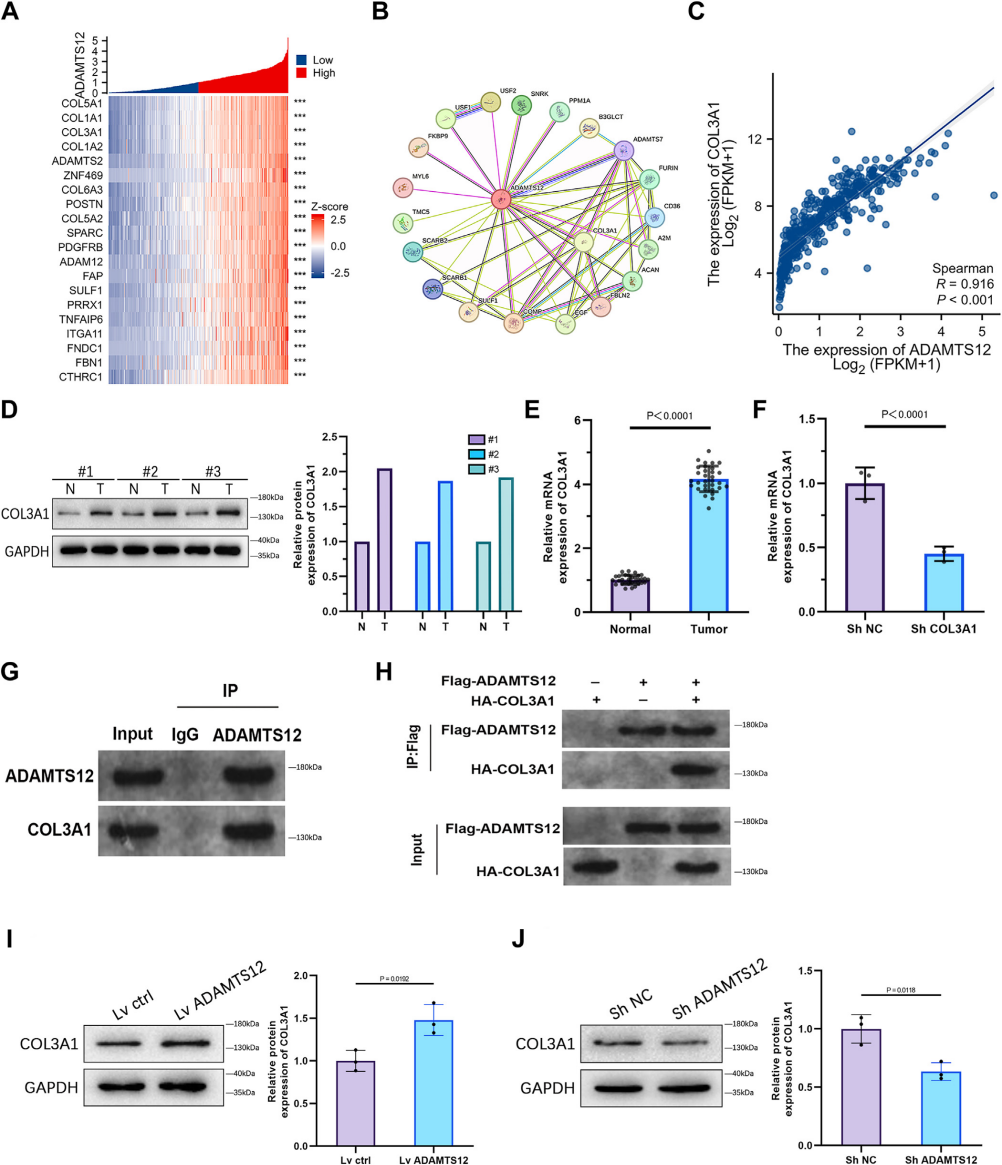
**Regulation of COL3A1 expression in BCa cells is mediated by ADAMTS12.***A*, the heatmap displays the 20 most prominent genes that demonstrate a strong positive correlation with ADAMTS12 in BCa. *B*, the identification of the interaction between ADAMTS12 and COL3A1 was accomplished through PPI analysis utilizing the STRING database. *C*, the expression of COL3A1 and ADAMTS12 showed a strong positive connection (Spearman's correlation coefficient=0.916). *D* and *E*, the levels of COL3A1 in BCa samples were found to be upregulated compared to that in adjacent normal tissues, as demonstrated by Western blotting (n = 3) and qRT-PCR analysis (n = 31). *F*, COL3A1 knockdown impacts on T24 cells were evaluated using qRT-PCR analysis for validation purposes (n = 3). *G*, the endogenous Co-IP method was utilized to validate the physical interactions between ADAMTS12 and COL3A1 in T24 cells. *H*, the Co-IP technique was employed to characterize the protein-protein interactions between exogenously expressed ADAMTS12 and exogenously expressed COL3A1 in HEK293T cells. *I* and *J*, the Western blot analysis was conducted to assess the COL3A1 expression in T24 cells after manipulating ADAMTS12 through overexpression or knockdown (n = 3 respectively). The data are presented as the mean ± SD. Unpaired t-tests were employed to compare two groups. ∗*p* < 0.05; ∗∗∗*p* < 0.001.

To improve our understanding of the potential biological functions of ADAMTS12 in BCa, we executed co-expression analysis utilizing data from the TCGA-BLCA dataset. Heatmaps were generated to visually represent the top 20 genes that showed a significant positive connection with ADAMTS12 in BCa (Fig. 7*A*). To get an in-depth understanding of the biological processes connected with ADAMTS12, we employed the STRING database to produce a protein-protein interaction (PPI) network that particularly focuses on ADAMTS12 in BCa. Interestingly, our analysis revealed a potential relationship between ADAMTS12 and COL3A1 (Fig. 7*B*). We examined the link between ADAMTS12 and COL3A1 expression levels in TCGA-BLCA patient data, a significant positive connection was identified between the two genes in BCa (Fig. 7*C*). COL3A1 expression levels were significantly raised in the tissues of BCa in contrast to their corresponding healthy tissues, as demonstrated by Western blotting and qRT-PCR analyses (Fig. 7, *D* and *E*). We successfully generated T24 cell lines with stable COL3A1 expression downregulation, which was verified by qRT-PCR analysis to validate the knockdown efficiency (Fig. 7*F*). Consequently, we proceeded to validate the direct connection between ADAMTS12 and COL3A1. The validation of this interaction was conducted through endogenous Co-IP assays in T24 cells (Fig. 7*G*) and exogenous Co-IP assays in HEK293T cells (Fig. 7*H*). The Western blot analysis revealed a direct link between increased ADAMTS12 expression and elevated COL3A1 levels in T24 cells (Fig. 7*I*). In contrast, decreased ADAMTS12 expression was linked with COL3A1 suppression in T24 cells (Fig. 7*J*).

### ADAMTS12 suppresses cellular apoptosis while promoting cell proliferation, invasion, and FAK/PI3K/AKT pathway activation through its interaction with COL3A1 in BCa cells

**Figure 8:**
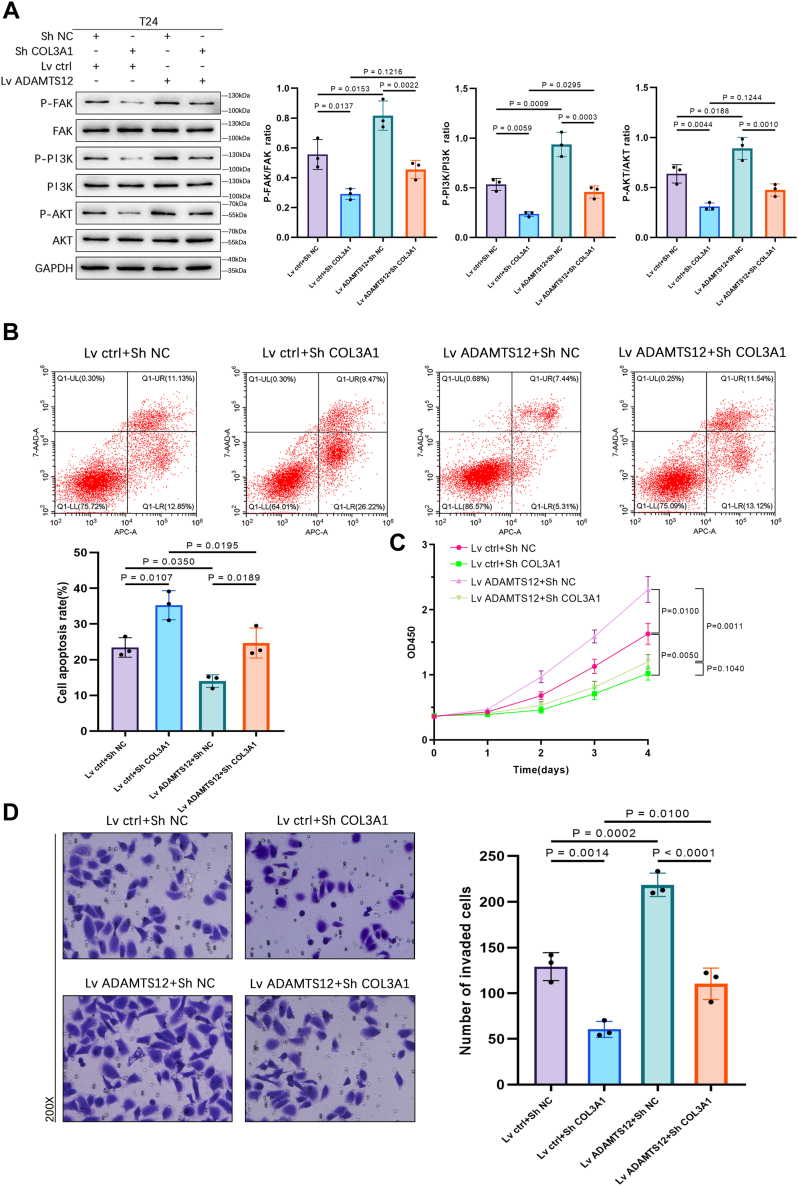
**ADAMTS12 suppresses cellular apoptosis while promoting cell growth, invasion, and activation of the FAK/PI3K/AKT pathway through its interaction with COL3A1 in BCa cells.***A*, Western blot analysis was conducted to assess the expression levels of p-FAK, p-PI3K, FAK, PI3K, p-AKT, and AKT proteins in T24 cells following the introduction of ADAMTS12 overexpression and/or COL3A1 knockdown (n = 3 respectively). *B*, the apoptotic process of T24 cells was evaluated by flow cytometry following transfection (n = 3). *C*, CCK-8 assay was utilized to assess T24 cells growth subsequent to transfection (n = 3). *D*, Transwell assay was utilized to evaluate the invasive potential of T24 cells following transfection (n = 3). The data are presented as the mean ± SD. Unpaired t-tests were employed to compare the two groups. One-way ANOVAs with the Tukey test were used for comparisons involving more than two groups. ∗*p* < 0.05.

Western blot analysis exhibited that COL3A1 inhibition efficiently attenuated the enhanced phosphorylation levels of FAK, PI3K, and AKT induced by ADAMTS12 overexpression (Fig. 8*A*). Flow cytometry results revealed that the suppressive impact of ADAMTS12 overexpression on cellular apoptosis was abrogated upon COL3A1 silencing (Fig. 8*B*). Cell proliferation enhancement induced by ADAMTS12 elevation was stopped upon COL3A1 silencing, as detected by the CCK-8 assay (Fig. 8*C*). The enhanced cellular invasion caused by the ADAMTS12 overexpression was shown to be reversed when COL3A1 was silenced in the Transwell assay (Fig. 8*D*).

### ADAMTS12 promotes tumor growth and triggers FAK/PI3K/AKT signaling pathway activation in BCa *in vivo*

**Figure 9:**
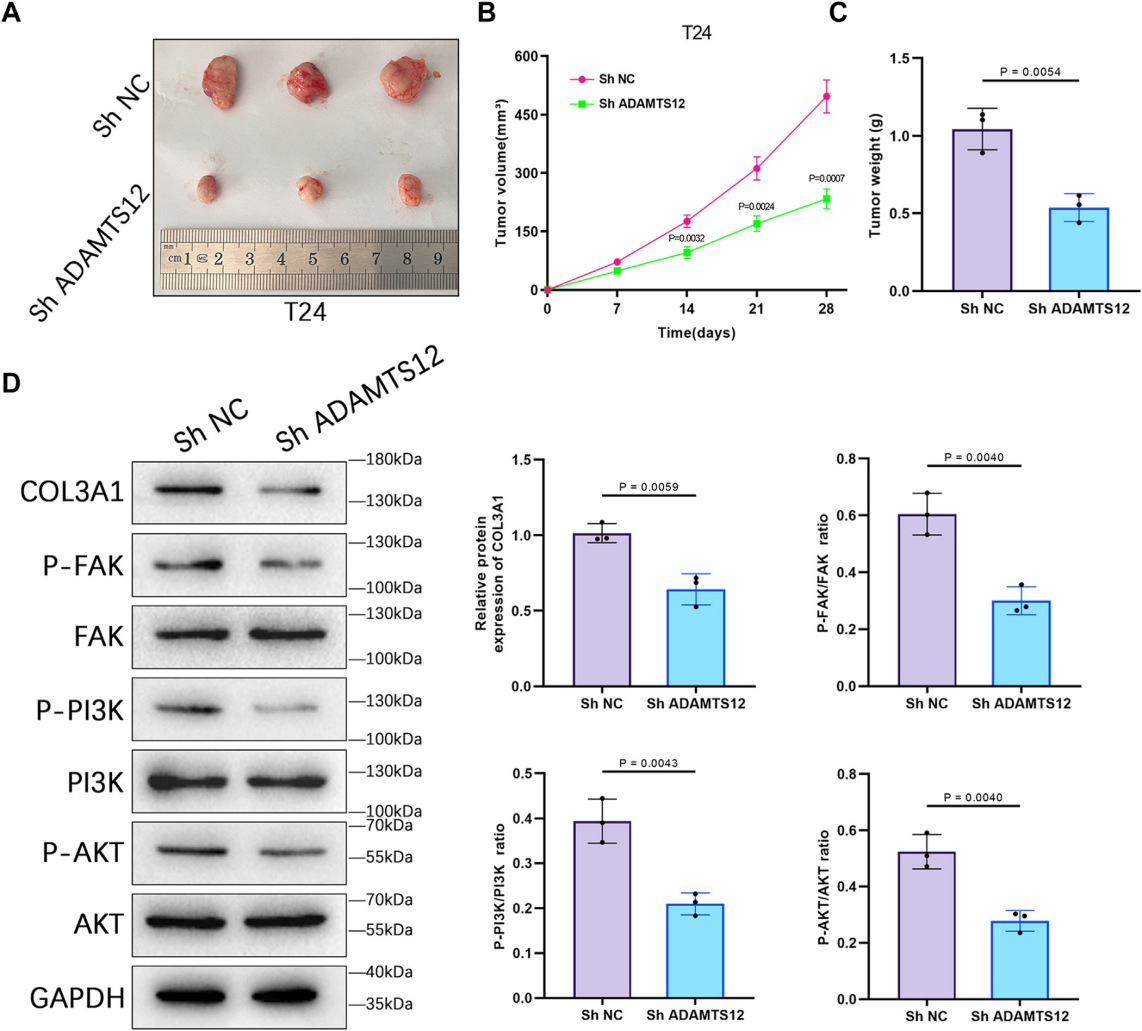
**ADAMTS12 promotes tumor growth and triggers FAK/PI3K/AKT signaling pathway activation in BCa *in vivo*.***A*, the T24 cells transfected with shNC or shADAMTS12 were used to generate subcutaneous xenograft tumors. *B*, the xenograft tumor volumes in the shNC group and shADAMTS12 group were measured (n = 3 respectively). *C*, the weights of xenograft tumors in the shNC group and shADAMTS12 group were measured (n = 3 respectively). *D*, the expression levels of COL3A1, p-FAK, FAK, p-PI3K, PI3K, p-AKT, and AKT were determined in the BCa xenograft tissues by Western blotting analysis (n = 3 respectively). The data are presented as the mean ± SD. Unpaired t-tests were employed to compare two groups. ∗*p* < 0.05.

ADAMTS12's influence on BCa growth *in vivo* was investigated by establishing xenograft models for *in vivo* experimentation. Downregulation of ADAMTS12 caused significant suppression of the growth rate, volume, and weight of xenograft tumors *in vivo* (Fig. 9, *A*–*C*). Subsequently, the expression levels of COL3A1, p-FAK, FAK, p-PI3K, PI3K, p-AKT, and AKT in the BCa xenograft tissues were determined through Western blotting analysis. The downregulation of ADAMTS12 significantly reduced the expression levels of p-PI3K, COL3A1, p-FAK, and p-AKT. Nevertheless, it had no significant effect on the levels of PI3K, FAK, and AKT. (Fig. 9*D*).

## Discussion

BCa is a prevalent malignant neoplasm with a significant propensity for progression, profoundly impacting individuals' quality of life (14). There is an increasing body of research on BCa indicating that alterations in molecular mechanisms have a crucial function in the progression of this malignant condition (15). In recent years, researchers have identified many prognostic and diagnostic biomarkers in bladder cancer. The FGFR3 gene, in particular, has been recognized as an actionable target in bladder cancer, with mutations commonly associated with favorable outcomes in non-invasive cases (16). Studies have shown that FGFR3 mutations correlate with lower tumor grades and stages, suggesting that patients with these mutations may benefit more from targeted therapies (17). HER2, another significant biomarker, has been implicated in bladder cancer prognosis. Its overexpression can provide insights into tumor aggressiveness and may guide treatment decisions, especially in higher-risk patients (18). To ensure early detection and prompt treatment of bladder malignancy, it is imperative to precisely detect the appropriate molecular targets.

ADAMTSs, a group of proteases released by malignant cells or nearby stromal cells, primarily exist in the extracellular matrix (ECM) and function as modulators of the tumor microenvironment (TME) (19). ADAMTS12, an ADAMTS protease family member, has been observed before to exert an impact on the progression of various malignancies, including gastric, pancreatic, and cervical cancers and head and neck squamous cell carcinoma (9, 12, 13, 20). Hou *et al.* (21) proposed that ADAMTS12 may potentially facilitate the progression of gastric malignancy and contribute to modifications in both TME and metabolic alterations within the tumor. The study conducted by Zou *et al.* demonstrates a significant connection between the ADAMTS12 gene and the migratory and invasive capabilities of cervical cancer, suggesting its potential as a predictive marker for subjects with poor outcomes (20). Liang *et al.* (22) demonstrated that miR-186 acts as a tumor suppressor in BCa by binding to ADAMTS12 *in vitro*. Xiong *et al.* (23) showed that the overexpression of LINC01929 promotes BCa development and elucidates the molecular mechanism by which advanced BCa highly expresses LINC01929, upregulating ADAMTS12 expression through competitive adsorption of miR-6875-5p. However, the precise mechanism of action of ADAMTS12 in bladder cancer remains elusive, necessitating further research. In our investigation, we detected that ADAMTS12 expression was upregulated and demonstrated a positive relationship with poor OS, DSS, and PFI in BCa. Moreover, we investigated the biological functions of ADAMTS12 and elucidated its pro-proliferative, anti-apoptotic, pro-metastatic and pro-invasive properties in BCa cells.

A cytoplasmic protein tyrosine kinase, focal adhesion kinase (FAK), governs the movement of the cytoskeleton and plays an indispensable role in cellular locomotion (24). FAK has a pivotal function in mediating the transduction of signals from integrins, thereby facilitating the activation of both PI3K and AKT (25). The PI3K/AKT pathway is aberrantly activated in many malignancies, and the dysregulated activation of this signaling cascade is associated with augmented proliferation, invasion, migration of neoplastic cells, as well as acquisition of drug resistance (26). Previous studies have elucidated the pivotal function of FAK/PI3K/AKT pathway activation in the BCa initiation and progression (27, 28). In this investigation, we conducted GO, KEGG, and GSEA analyses and identified a noteworthy correlation between increased ADAMTS12 expression and the activation of focal adhesion as well as the PI3K/AKT pathway. Hence, we conducted further investigations to elucidate the protein expression patterns associated with the pathway of FAK/PI3K/AKT *in vitro* and *in vivo*. The FAK/PI3K/AKT pathway activation was observed upon overexpression of ADAMTS12, while suppression of ADAMTS12 resulted in FAK/PI3K/AKT pathway suppression in BCa cells. Hence, our outcomes suggest that ADAMTS12 facilitates the FAK/PI3K/AKT pathway activation, thereby having a function in the development of aggressive phenotypes in BCa.

Collagen type III alpha one chain (COL3A1), a crucial component of the ECM proteins, has a pivotal function in maintaining the structural integrity of multiple organs and contributes to processes such as fibrosis and tissue regeneration (29). Tumor-associated fibroblasts have been implicated in the cancer microenvironment, with certain molecules (such as COL3A1) produced by these cells being associated with tumorigenesis and metastasis. Consequently, they have emerged as potential targets for precision therapy in cancer treatment (30). The expression of COL3A1 is significantly upregulated in breast, gastric, head and neck, and colorectal cancers, underscoring its potential as a prognostic predictor for these types of cancer (31, 32, 33, 34). Van Espen *et al.* (35) discovered that RNF185-mediated abnormal expression of COL3A1 exerts a potent regulatory influence on the metastatic potential of prostate cancer. Additionally, in triple-negative breast cancer, COL3A1 suppression causes a decrease in PD-L1 expression and subsequently inhibits cancer cell proliferation and metastasis (36). To enhance our comprehension of the molecular pathways that are connected with the advancement of BCa through ADAMTS12, we have discovered an interaction between ADAMT12 and COL3A1, suggesting a positive connection between ADAMTS12 and COL3A1 in BCa. Moreover, the inhibition of COL3A1 led to a reversal of the observed enhancement in cell growth and invasion caused by ADAMTS12 overexpression, as well as a suppression of cell apoptosis.

In summary, our investigation demonstrates that ADAMTS12 acts as an oncogene by enhancing cell growth, invasion, and migration while suppressing the apoptotic process in BCa through modulation of the FAK/PI3K/AKT signaling pathway *via* regulation of COL3A1. Collectively, these findings provide compelling evidence supporting the role of ADAMTS12 as a valued indicator for both diagnosis and prognosis prediction in BCa, thereby offering potential insights into a novel therapeutic approach for BCa.

## Experimental procedures

### Database

The Cancer Genome Atlas (TCGA) database, available at https://portal.gdc.cancer.gov, was employed to acquire publicly available RNA sequencing data and survival statistics of individuals with BCa. For the analysis of Cox proportional hazards models, we obtained survival data from the TCGA-BLCA dataset, which comprises 411 BCa samples. To explore potential protein-protein interactions, the potential associations between ADAMTS12 and other proteins were investigated using the database of STRING (https://string-db.org/).

### Bioinformatics

Differentially expressed genes (DEGs) data were obtained using the DESeq2 package, calculated in terms of Fragment Per Kilobase of transcript per Million mapped reads (FPKM) values. The survival package was utilized to do an evaluation of hypotheses on proportional hazards and conduct survival regression analysis. In addition, produced findings were visualized employing survminer and ggplot2 packages. The pROC package in R was employed to create the curve of receiver–operating characteristic (ROC) for diagnostic purposes. The correlation measurements were evaluated utilizing Spearman's rank correlation coefficient, employing the cor.test package in R. Enrichment analyses for Gene Ontology (GO), Kyoto Encyclopedia of Genes and Genomes (KEGG), and Gene Set Enrichment Analysis (GSEA) were conducted employing the clusterProfiler package in R.

### Clinical samples

Typically, 31 collections of BCa and their corresponding neighboring healthy tissues were obtained from subjects who had BCa and had radical cystectomy at Renmin Hospital of Wuhan University between 2019 and 2022. The tissues promptly underwent liquid nitrogen freezing. The research protocol received approval from the Institutional Review Board at Renmin Hospital of Wuhan University and adhered to the principles of the Declaration of Helsinki. Every participant in this study gave written consent after receiving comprehensive information. The clinicopathological characteristics of the 31 cases diagnosed with BCa were thoroughly documented and recorded in Table 1.

**Table 1:** Clinicopathological features between the two groups of ADAMTS12

Characteristics	Cases (n = 31)	ADAMTS12 expression		*p*-value
		Low (n = 15)	High (n = 16)	
Age				0.458
≤65	10	6	4	
>65	21	9	12	
Gender				0.685
Female	8	3	5	
Male	23	12	11	
T stage				0.037^a^
T1-2	7	6	1	
T3-4	24	9	15	
N stage				0.003^a^
N0	18	13	5	
N1-3	13	2	11	
M stage				0.484
M0	29	15	14	
M1	2	0	2	

a *p* < 0.05 considered statistically significant.

### Cell culture and transfection

HEK-293T, T24, UMUC3, and 5637 cell lines, in addition to the normal urothelial SV-HUC-1 cell line, were acquired from the American Type Culture Collection (ATCC). The HEK-293T, SV-HUC-1 and T24 cell lines were cultivated in DMEM media, whilst RPMI 1640 medium was employed to maintain the 5637 and UMUC3 cells. The culture plates were placed in a controlled humidity room and underwent incubation at 37 °C, with the addition of 5% CO_2_. The media and supplementation employed in this investigation were acquired from Beyotime Biotechnology. The basic medium was treated with 10% FBS and 1% penicillin/streptomycin (Beyotime).

The lentiviruses purchased from GeneChem were utilized for the overexpression and knockdown of ADAMTS12 and COL3A1. The T24 and 5637 cells underwent transfection with lentivirus employing HiTransG P infection-enhancing solution at an MOI of 10. Next, quantitative real-time PCR (qRT-PCR) analysis and Western blot were employed to identify and confirm the cellular effectiveness of transfection using puromycin (2 μg/ml).

### Western blot assay

The Bicinchoninic Acid protein assay (BCA; Beyotime) was employed to isolate and detect all of the protein in the cell lines or tissues. Typically, 10% SDS polyacrylamide gels from Solarbio were employed to perform protein isolation which then underwent transferring onto PVDF membranes from Bio-Rad. The membranes underwent 60 min of blockage at room temperature with a solution consisting of PBS and 5% nonfat milk. Subsequently, the membranes underwent overnight incubation with primary antibodies targeting ADAMTS12 (ab45041, Abcam), p-AKT (ab38449, Abcam), COL3A1 (ab184993, Abcam), FAK (ab40794, Abcam), p-FAK (ab81298, Abcam), p-PI3K (ab182651, Abcam), AKT (ab8933, Abcam), PI3K (ab302958, Abcam), GADPH (ab8245, Abcam) at 4 °C. After that, three washes with PBST were conducted to membranes which were then, at room temperature, exposed for 1 h to a horseradish peroxidase-conjugated secondary antibody. The enhanced chemiluminescence (ECL) technique was utilized to detect the signals subsequent to incubation with the secondary antibody.

### RNA isolation and qRT-PCR

The TRIzol reagent (Invitrogen) was utilized for obtaining total RNA from human cells or tissues depending on the directions provided by the manufacturer. Following that, the first cDNA strand was created employing a reverse transcription system kit (Vazyme). The ChamQ Universal SYBR qPCR Master Mix from Vazyme in China and the CFX96 Real-Time System from Bio-Rad in the USA were employed to conduct the qRT-PCR procedure. The parameters of qPCR were as stated: The procedure begins with an initial denaturation phase for 30 s at 95 °C. This is then followed by 40 rounds of amplification, with each one consisting of 10 s at 95 °C and 30 s at 60 °C. GAPDH was employed as a control variable in this experiment. Subsequently, the relative fold change was determined by employing the 2^−ΔΔCT^ technique. The sequences of primer are as follows: ADAMTS12 Forward: 5′-GCCATGGACTGACTGGATTT-3′, Reverse: 5′-TGCCTCCTGTAAACGATGTG-3′; COL3A1 Forward: 5′-GCTCTGCTTCATCCCACTATTA-3′, Reverse: 5′-CTGGCTTCCAGACATCTCTATC-3′; GAPDH Forward: 5′-GTGGACCTGACCTGCGTCT-3′, Reverse: 5′-GTGTCGCTGTTGAAGTCAGAGGAG-3′.

### Cell proliferation assay

Cell Counting Kit-8 (CCK-8; Dojindo, Japan) was employed to examine the viability and growth of the cell. For the CCK-8 assay and at a density of 1000 cells per well, 96-well plates were utilized for the seeding of BCa cells. Depending on the producer's guidelines, 10 ml of CCK-8 solution was supplemented to the wells and incubated for a duration of 3 h. Subsequently, the optical density was identified at 450 nm.

### Cell apoptosis assay

The samples underwent a washing step followed by trypsinization in the absence of EDTA. Then, the cells underwent two centrifugation rounds and were rinsed with pre-chilled PBS. The cells were then gently resuspended in 1 × Binding Buffer that had been pre-cooled was employed to resuspend the cell. Following the manufacturer's directions, to evaluate cell apoptosis, we utilized the Annexin V-FITC/PI Cell Apoptosis Detection Kit (Servicebio). Finally, a flow cytometer was utilized to measure the apoptotic rate of the cell.

### Wound healing assay

To start the process of wound healing, we put cells into 6-well plates with moderate features. The goal of the cell implementation was to accomplish a rate of fusion of 100% following an incubation period of overnight. A sterile pipette tip of 100 μl was utilized to delicately extract the cell layer from the interior of the well, causing the formation of a linear gap. The wounds were evaluated instantly following the scrape and then reevaluated a day later. The areas without structures of cells and the rest of the areas were identified by the analysis of digital photographs.

### Transwell assay

The ability of transfected BCa cells to invade was assessed employing a Transwell chamber (with a size of the pore of 8.0 μm; manufactured by Millipore) that was covered with Matrigel (produced by BD Biosciences). Concisely, the top section was loaded with 100 μl of a suspension of cells in DMEM without FBS, while the inferior section was loaded with 600 μl of DMEM with 10% FBS. Subsequent to a 2-day incubation period, the cells in the lower chamber were subjected to methanol treatment for preservation and then underwent crystal violet staining. The cells that were stained were then quantified under an optical microscope.

### Coimmunoprecipitation (Co-IP) assay

The exogenous Co-IP assay was conducted through co-transfection of Flag-ADAMTS12 and HA-COL3A1 into HEK-293T cells, followed by cell lysis using an IP buffer containing 20 mM Tris-HCl, 1 mM EDTA, 150 mM NaCl, and 1% NP-40. The buffer solution was supplemented with a 1% phosphatase inhibitor cocktail. The lysis process was carried out at 4 °C for 30 minutes. After centrifugation at 12,000*g* and 4 °C for 10 minutes, the supernatant was collected. Subsequently, it was incubated overnight at 4 °C with magnetic beads (20 μl) and antibodies (Flag-tag and HA-tag, each measuring approximately 2–3 μl). The next day, the supernatant was extracted using a magnetic stand. The beads responsible for binding a specific antibody and the target protein were then reconstituted in 1 × loading buffer and subjected to denaturation at 95 °C for 10 min. Subsequently, the specimens were centrifuged at a speed of 12,000*g* for 1 to 2 min at a temperature of 4 °C. Following this step, the resulting supernatant was carefully preserved at −80 °C in preparation for immunoblot analysis. The endogenous Co-IP assay was performed in T24 cells transfected with Flag-ADAMTS12 using either the Flag antibody or IgG, following identical procedures as the exogenous Co-IP assay.

### *In vivo* experiment

The animal investigations were performed depending on the standards set out by the Institutional Animal Care and Use Ethics Committee of Renmin Hospital of Wuhan University. Four-week-old male BALB/c nude mice were acquired from WQJX BioTechnology in Wuhan, China. Following a period of 7-day acclimatization, the nude mice were assigned to their respective groups at random, each consisting of three individuals. In the model of xenograft, T24 cells that had undergone stable transfection were subjected to suspension in 200 μl of PBS and then administered subcutaneously into the nude mice flanks. The size of the tumor, including its length and width (a and b), was determined by employing a Vernier caliper at intervals of 7 days. The volumes of tumors were detected by applying the formula ab^2^/2. At the end of the fourth week, upon sacrificing the mice, tumor nodules were obtained, and their weights were determined.

### Statistical analysis

GraphPad Prism 8.0 was employed to apply statistical analysis. The data obtained from three independent experiments were presented as mean ± standard deviation (SD). The variations between two groups were examined employing two-tailed unpaired Student's *t* test, while a one-way analysis of variance, accompanied by Tukey's multiple comparisons test, was performed to determine variations across several groups. Statistical significance was considered as any difference that had a *p*-value less than 0.05.

## Data availability

The data used and analyzed during the current study are available from the corresponding author on reasonable request.

## Ethics statement

All procedures were done according to protocols approved by the Clinical Research Ethics Committees of Renmin Hospital of Wuhan University and conducted in accordance with the guidelines of ethical management. Committee approval number: WDRY2019-K035. All animal experiments conducted was compliant with the Ethical Committee of Renmin Hospital of Wuhan University.

## Supporting information

This article contains supporting information.

## Conflict of interest

The authors declare that they have no conflicts of interest with the contents of this article.
