# Supplementary material for: Unveiling ADAMTS12: A key driver of bladder cancer progression via COL3A1-Mediated activation of the FAK/PI3K/AKT signaling pathway
Source: J Biol Chem. 2025 Jan 4;301(2):108155. doi: 10.1016/j.jbc.2025.108155 (PMC11795591; doi:10.1016/j.jbc.2025.108155)
Supplement: Supporting information [file mmc1.docx]

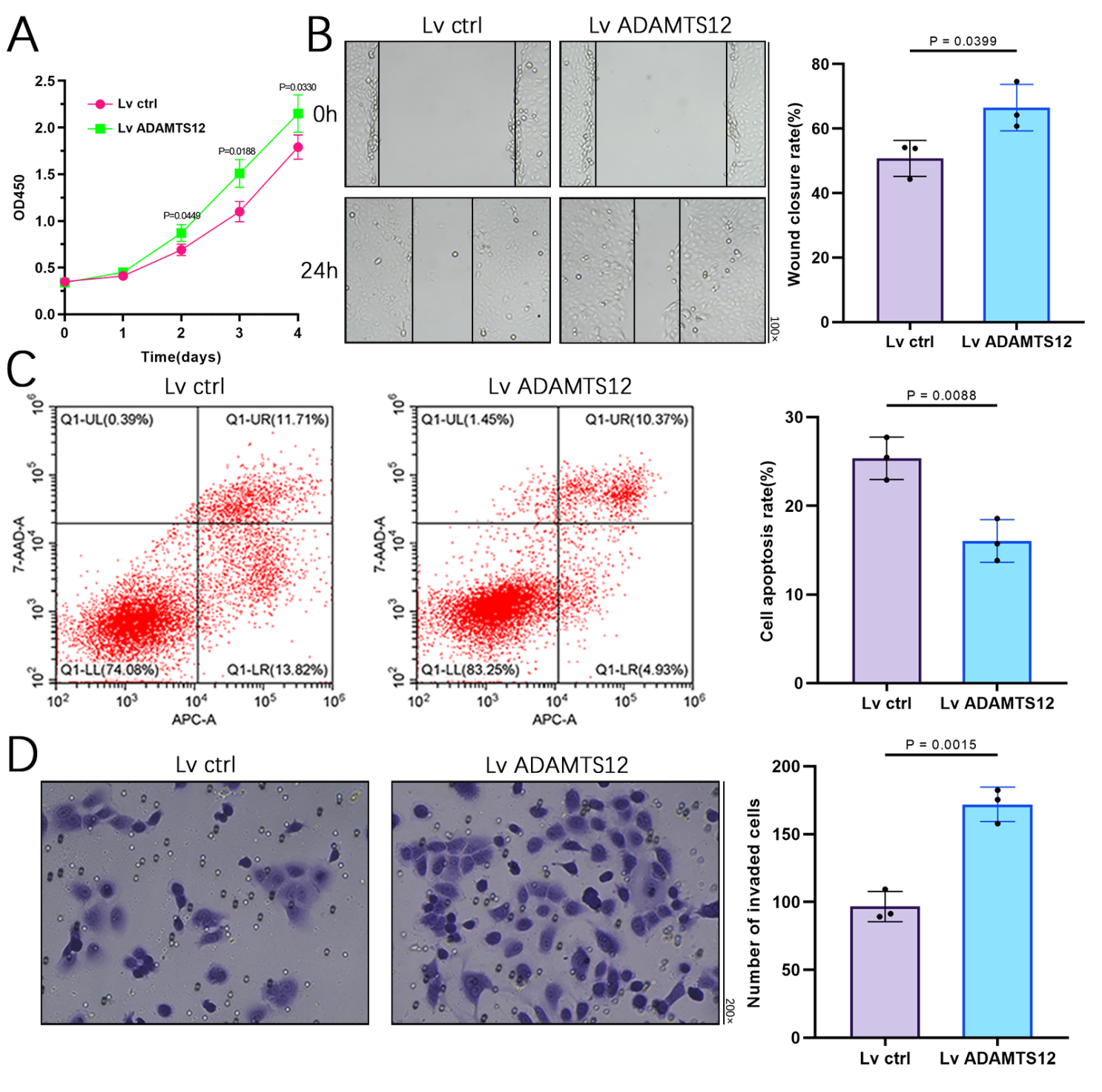


**Fig. S1 The overexpression of ADAMTS12 suppresses apoptosis and promotes the in vitro proliferation, migration, and invasion of 5637 cells.** (A) 5637 cell viability was examined employing the CCK-8 assay subsequent to overexpression of ADAMTS12 (n = 3). (B) Wound-healing assay demonstrated that the upregulation of ADAMTS12 expression in 5637 cells promoted cell migration (n = 3). (C) The apoptotic rate of 5637 cells was assessed by flow cytometry following overexpression of ADAMTS12 (n = 3). (D) Transwell assay demonstrated that ADAMTS12 overexpression in 5637 cells significantly enhanced invasion (n = 3). The data are presented as the mean ± SD. Unpaired t-tests were employed to compare two groups.
